# Live-Cell Monitoring and Transcriptomic Profiling Identify Distinct Temporal Patterns of Cell-Cycle Activity and Maturation in Human iPSC-Derived Cardiomyocytes

**DOI:** 10.3390/cells15211936

**Published:** 2026-10-24

**Authors:** Shiqiao Ye, Rabina Shrestha, Yang Yu, Matthew Alonzo, Cankun Wang, Qin Ma, Ming-Tao Zhao

**Affiliations:** 1 Center for Cardiovascular Research, Abigail Wexner Research Institute, Nationwide Children’s Hospital, Columbus, OH 43205, USA; 2 The Heart Center, Nationwide Children’s Hospital, Columbus, OH 43205, USA; 3 Department of Biomedical Informatics, The Ohio State University College of Medicine, Columbus, OH 43210, USA; 4 Pelotonia Institute for Immuno-Oncology, The James Comprehensive Cancer Center, The Ohio State University, Columbus, OH 43210, USA; 5 Department of Pediatrics, The Ohio State University College of Medicine, Columbus, OH 43210, USA; 6 Dorothy M. Davis Heart and Lung Research Institute, The Ohio State University Wexner Medical Center, Columbus, OH 43210, USA

**Keywords:** human iPSC-derived cardiomyocytes, cardiomyocyte prolifération, cardiomyocyte maturation, FUCCI reporter, cell cycle

## Abstract

Human induced pluripotent stem cell-derived cardiomyocytes (hiPSC-CMs) show decreasing cell-cycle activity during prolonged culture. However, it remains unclear whether changes in cell-cycle activity occur before, after, or concurrently with transcriptional changes in cell-cycle and maturation-associated programs. Here, we combined a TNNT2-FUCCI reporter, Ki67 immunostaining, and bulk RNA sequencing to define how cell-cycle activity changes during prolonged human iPSC-cardiomyocyte culture. FUCCI and Ki67 analyses showed similar levels of cell-cycle activity at days 20 and 30 of cardiomyocyte differentiation but declined more clearly by day 40. In contrast, transcriptomic profiling revealed that canonical cell-cycle and DNA-replication programs were already reduced by day 30. The subsequent day-30-to-day-40 interval was characterized by increased expression of sarcomeric, contractile, and calcium-handling gene programs rather than a further broad loss of mitotic pathways. These findings support a staged model of human iPSC-cardiomyocyte maturation-associated transcriptional remodeling in which a reduction in cell-cycle-associated transcription is detectable before the subsequent decline in cardiomyocyte cell-cycle activity, thereby identifying distinct temporal patterns of cell-cycle and maturation-associated changes during prolonged hiPSC-CM culture.

## 1. Introduction

Human induced pluripotent stem cell-derived cardiomyocytes (hiPSC-CMs) have become an important platform for disease modeling, drug discovery, and studies of cardiac regeneration [1,2]. Their utility continues to expand as differentiation and culture methods improve, yet the relatively immature state of hiPSC-CMs remains a well-recognized limitation. Prolonged culture can enhance structural, metabolic, electrophysiological, and functional maturation, but this process is also associated with reduced proliferative competence [3,4,5]. Defining how proliferation changes during maturation is therefore important both for understanding cardiomyocyte biology and for identifying developmental windows during which proliferative potential may still be retained.

Reduced proliferative activity is generally associated with cardiomyocyte maturation and postnatal cell-cycle restriction, but the temporal sequence of events underlying this transition remains incompletely understood [6,7,8]. One possibility is that proliferation decline occurs in parallel with suppression of cell-cycle and DNA-replication programs. Another possibility is that cardiomyocytes pass through an intermediate stage in which transcriptional withdrawal from the cell cycle begins before a decline in measurable cell-cycle activity becomes evident. Distinguishing between these possibilities would refine current models of hiPSC-CM maturation and may help define a proliferation-permissive window before maturation-linked restriction is fully established.

Reporter-based approaches have provided valuable tools for examining cardiomyocyte cell-cycle dynamics, including in human iPSC-derived cardiomyocytes, and have expanded the ability to study proliferation in real time [6,7,8,9]. The fluorescent ubiquitination-based cell-cycle indicator (FUCCI) system uses fluorescently tagged, cell-cycle-regulated proteins that undergo phase-dependent degradation, allowing cell-cycle state to be visualized in living cells [9]. When coupled to a cardiomyocyte-specific TNNT2 promoter, this approach enables lineage-specific visualization of cell-cycle dynamics, with nuclear mKO2 fluorescence marking G1 and mAG/Clover fluorescence marking S/G2/M phases [10]. This cardiomyocyte-specific reporter therefore enables visualization of cell-cycle state without requiring cell fixation. These systems have been particularly useful for visualizing cell-cycle dynamics and identifying conditions that influence proliferative behavior. However, the temporal relationship between changes in cell-cycle activity and broader maturation-associated transcriptional remodeling remains less well defined in staged human iPSC-CM culture models.

In the present study, we combined a TNNT2-FUCCI reporter line, Ki67 immunostaining, and bulk RNA sequencing to examine cell-cycle activity and maturation-associated transcriptional changes across days 20, 30, 40, and 50 of human iPSC-cardiomyocyte culture. We found that FUCCI- and Ki67-defined cell-cycle activity was not significantly altered at days 20 and 30, despite reduction in cell-cycle and DNA-replication programs by day 30. Instead, the subsequent interval between day 30 and day 40 was characterized by increased expression of sarcomeric, myofibrillar, and calcium-handling programs, coincident with a decline in cell-cycle activity. Together, these findings support a staged pattern of hiPSC-CM transcriptional remodeling and suggest an intermediate window of sustained cell-cycle activity before its subsequent decline and the increased expression of maturation-associated transcriptional programs.

## 2. Methods

### 2.1. Generation of Patient-Derived iPSCs

Patient-specific induced pluripotent stem cells (iPSCs) were generated from peripheral blood mononuclear cells (PBMCs) using the CytoTune-iPS 2.0 Sendai Reprogramming Kit (Thermo Fisher Scientific, Waltham, MA, USA) according to the manufacturer’s instructions. Briefly, PBMCs were cultured for approximately 1 week in the complete StemPro-34 medium before transduction with Sendai viral vectors encoding KLF4, OCT3/4, SOX2, and c-MYC. After overnight incubation, cells were transferred to Matrigel-coated plates and maintained in fresh complete StemPro-34 medium. After 48 h, the medium was changed to cytokine-free StemPro-34 medium for 2 days, followed by daily medium changes with the complete Essential 8 (E8) medium. Emerging iPSC colonies were manually picked, expanded on Matrigel-coated plates, and clonally purified over several weeks [11].

### 2.2. CRISPR/Cas9-Mediated Knock-In of the TNNT2-FUCCI Reporter

Genome editing was performed in healthy patient-derived iPSCs using CRISPR/Cas9-mediated homologous recombination at the AAVS1 safe-harbor locus. When cultures reached approximately 70–80% confluence, cells were rinsed with DPBS and incubated with 0.5 mM EDTA for 5 min at 37 °C. Cells were gently dissociated in the E8 medium supplemented with ROCK inhibitor, counted, and pelleted by centrifugation. A total of 2 × 10^6^ cells were resuspended in 100 µL of P3 nucleofection solution supplemented with 1.5 µg AAVS1 gRNA plasmid (Addgene #41818), 2.25 µg Cas9-GFP plasmid (Addgene #44719), and 3 µg AAVS1-Puro cTnT-FUCCI donor plasmid (Addgene #136935) [10]. Cells were incubated in nucleofection solution for 5 min at room temperature and electroporated using program CB150 on the 4D-Nucleofector System (Lonza Biosciences, Walkersville, MD, USA). Immediately after nucleofection, cells were plated onto Matrigel-coated 6-well plates in complete E8 medium containing 10 µM ROCK inhibitor. When cultures reached near-confluence, puromycin selection (1 µg/mL) was applied for approximately 1 week. Surviving colonies were expanded and screened for correct reporter integration.

### 2.3. Isolation and Genotyping of Single-Cell FUCCI iPSC Clones

To establish monoclonal TNNT2-FUCCI iPSC lines, puromycin-resistant cells were dissociated and plated at low density (approximately 2000–3000 cells per well of a 6-well Matrigel-coated plate) in 2 mL of the complete E8 medium supplemented with a 4 µM ROCK inhibitor. Cells were cultured for approximately 10 days until individual colonies derived from single cells became apparent. Individual colonies were mechanically divided into two fractions: one fraction was used for genomic DNA extraction and genotyping, and the second fraction was transferred to a Matrigel-coated 24-well plate for continued expansion. Genomic DNA was extracted using QuickExtract DNA Extraction Solution (Lucigen Corporation, Middletown, WI, USA: catalog no QE09050). PCR-based genotyping was performed using the primer pair F1 (5′-GAGAGAGATGGCTCCAGGAA-3′) and R1 (5′-CTGTTTCCCCTTCCCAGGCAGGTCC-3′), with an annealing temperature of 64 °C, to identify correctly targeted clones. An approximately 1000 bp PCR product was expected following successful targeting. To distinguish heterozygous and homozygous clones, PCR was additionally performed using F2 (5′-ATGTTTGCTGCCTCCAGGGATC-3′) and R2 (5′-ACGCGCGTGAGGAAGAGTTCTTGC-3′), with an annealing temperature of 60 °C. An approximately 400 bp product was expected for heterozygous clones. Correct integration of the TNNT2-FUCCI reporter at the AAVS1 locus was confirmed by Sanger sequencing (Eurofins Genomics, Louisville, KY, USA) and comparison of the resulting sequence with the expected AAVS1-FUCCI integration sequence. A single validated TNNT2-FUCCI iPSC clone derived from one donor iPSC line was used for all subsequent FUCCI cardiomyocyte differentiation and reporter-based experimental analyses in this study.

### 2.4. iPSC Maintenance and Cardiomyocyte Differentiation

iPSCs were maintained on Matrigel-coated plates in the complete E8 medium with daily medium changes. Cells were passaged when they reached approximately 90–95% confluence using DPBS and 0.5 mM EDTA. For cardiomyocyte differentiation, confluent iPSC cultures were treated with B27 minus insulin medium containing 6–10 µM CHIR for 2 days, followed by 1 day in fresh B27 minus insulin medium. Cells were then treated with B27 minus insulin medium containing 5 µM IWR for 2 days. Afterward, cells were maintained in B27 plus insulin medium. To promote metabolic selection and enrichment of cardiomyocytes, cultures were transferred to glucose-free B27 plus insulin medium for 3–4 days with medium changes every 2 days, followed by recovery in standard B27 plus insulin medium [11].

### 2.5. Live-Cell Imaging and Quantification of TNNT2-FUCCI Cardiomyocytes

Purified iPSC-derived cardiomyocytes were replated onto black-walled, glass-bottom 24-well imaging plates. Live-cell imaging was performed at days 20, 30, 40, and 50 after differentiation. Before imaging, the culture medium was replaced with a phenol red-free RPMI 1640/B27 medium. Images were acquired on a Leica THUNDER Imager using 10× or 20× objectives with FITC (488 nm) and Texas Red (594 nm) filter sets. Exposure settings were optimized to detect nuclear mAG and mKO2 signals corresponding to FUCCI reporter expression. Live images were analyzed using LAS X software, and nuclei positive for green or red fluorescence were quantified.

### 2.6. Immunofluorescence Staining, Imaging, and Quantification

For immunofluorescence analysis, iPSC-derived cardiomyocytes collected at days 20, 30, 40, and 50 were washed twice with DPBS and fixed with 4% paraformaldehyde for 15 min at room temperature. Additional day-19 cardiomyocytes were used for AURKB immunostaining to assess mitotic progression. Cells were washed with DPBS and permeabilized with 0.1% Triton X-100 in DPBS for 10 min at room temperature. Following two washes with DPBS, cells were blocked with 0.2% bovine serum albumin (BSA) in DPBS for 1 h at room temperature. Cells were incubated overnight at 4 °C with mouse monoclonal anti-Ki67 antibody (Invitrogen, Carlsbad, CA, USA; MA5-47446; 1:1000) and rabbit polyclonal anti-cardiac troponin T (TNNT2) antibody (Abcam, Cambridge, UK; ab45932; 1:1000) diluted in blocking solution. For AURKB analysis, day-19 cardiomyocytes were incubated overnight at 4 °C with anti-AURKB antibody (Abcam, ab315206; 1:1000) together with anti-cardiac troponin T (TNNT2) antibody (Abcam, ab45932; 1:1000). Following primary antibody incubation, cells were washed three times with blocking solution and incubated with goat anti-mouse IgG Alexa Fluor 594 and goat anti-rabbit IgG Alexa Fluor 488 secondary antibodies (Invitrogen; 1:2000) for 1 h at room temperature. Cells were subsequently washed three times with DPBS, and nuclei were counterstained with DAPI for 3–5 min at room temperature. Following a final wash, samples were mounted using SlowFade Gold Antifade Mountant (Thermo Fisher Scientific, Waltham, MA, USA).

Immunofluorescence images were acquired using a Leica THUNDER Imager. For each experimental condition, 10 randomly selected, non-overlapping fields were analyzed for each independent cardiomyocyte differentiation. Consistent image-acquisition settings were used across samples within each experiment. For FUCCI analysis, cardiomyocytes were classified based on mKO2 (red) and Clover (green) fluorescence. The percentage of Clover-positive FUCCI cardiomyocytes was calculated as the number of Clover-positive cardiomyocytes divided by the total number of FUCCI-positive cardiomyocytes analyzed, multiplied by 100. For Ki67 analysis, cardiomyocytes were identified by TNNT2 immunostaining, and Ki67-positive cardiomyocytes were defined as TNNT2-positive cells containing a Ki67-positive nucleus. The percentage of Ki67-positive cardiomyocytes was calculated as the number of Ki67-positive/TNNT2-positive cardiomyocytes divided by the total number of TNNT2-positive cardiomyocytes analyzed, multiplied by 100. Measurements from the 10 microscopic fields within each independent differentiation were combined to generate a single biological replicate value. Three independent cardiomyocyte differentiations were analyzed per time point (n = 3 biological replicates). Statistical analyses of FUCCI and Ki67 measurements were performed using GraphPad Prism 10 (GraphPad Software, Boston, MA, USA). Statistical significance was assessed using two-way ANOVA followed by Šídák’s multiple-comparisons test for pairwise comparisons between time points within each treatment condition. A *p*-value < 0.05 was considered statistically significant.

### 2.7. Bulk RNA Sequencing

Bulk RNA sequencing was performed on control iPSC-derived cardiomyocytes collected at days 20, 30, 40, and 50, with three independent cardiomyocyte differentiations per time point (n = 3). Total RNA was extracted from iPSC-derived cardiomyocytes at defined time points following medium removal and direct lysis in TRI Reagent (Zymo Research, Irvine, CA, USA). Lysates were stored at −80 °C until processing. RNA was purified using the Direct-zol RNA Miniprep Kit (Zymo Research, Irvine, CA, USA) according to the manufacturer’s instructions. RNA quantity was measured using a Qubit 4 Fluorometer and NanoDrop One spectrophotometer, and RNA integrity was assessed using an Agilent 2100 Bioanalyzer. For RNA sequencing, mRNA was isolated using oligo(dT) beads and fragmented. Complementary DNA (cDNA) was synthesized with random hexamer primers, followed by end repair, adaptor ligation, size selection, and PCR amplification to generate the cDNA library (Illumina, San Diego, CA, USA). This library was sequenced using paired-end sequencing on the Illumina NovaSeq 6000 system. Raw reads from bulk RNA-seq were processed to remove adapters using Cutadapt (version 3.5), and quality control was performed on the FASTQ files before and after this step using FASTQC software. Processed reads were then aligned to the GRCh38.p13 human reference genome using HISAT2 (version 2.2.1). SAM files generated were converted to BAM files, which were then sorted and indexed using SAMtools. Gene features were identified from the GENCODE GTF file, and counts per gene were determined using featureCounts (version 1.6.4). Data normalization and differential expression analysis were conducted using the DESeq2 R package, identifying genes with significant expression changes at a false discovery rate (FDR) of less than 0.05. Pathway analysis was conducted using gene set enrichment analysis (GSEA) with the “fgsea” package (version 1.22), adjusting for multiple testing using the Benjamini and Hochberg method. Gene sets with adjusted *p*-values less than 0.05 were considered significantly enriched. Volcano plots were generated using the ggplot2 R package (version 3.5.0). Differentially expressed genes were visualized if they met a threshold of adjusted *p*-value < 0.05 and an absolute log2 fold change (|logFC|) > 0.5.

## 3. Results

### 3.1. FUCCI and Ki67 Analyses Track Cardiomyocyte Cell-Cycle Activity During Maturation

**Figure 1:**
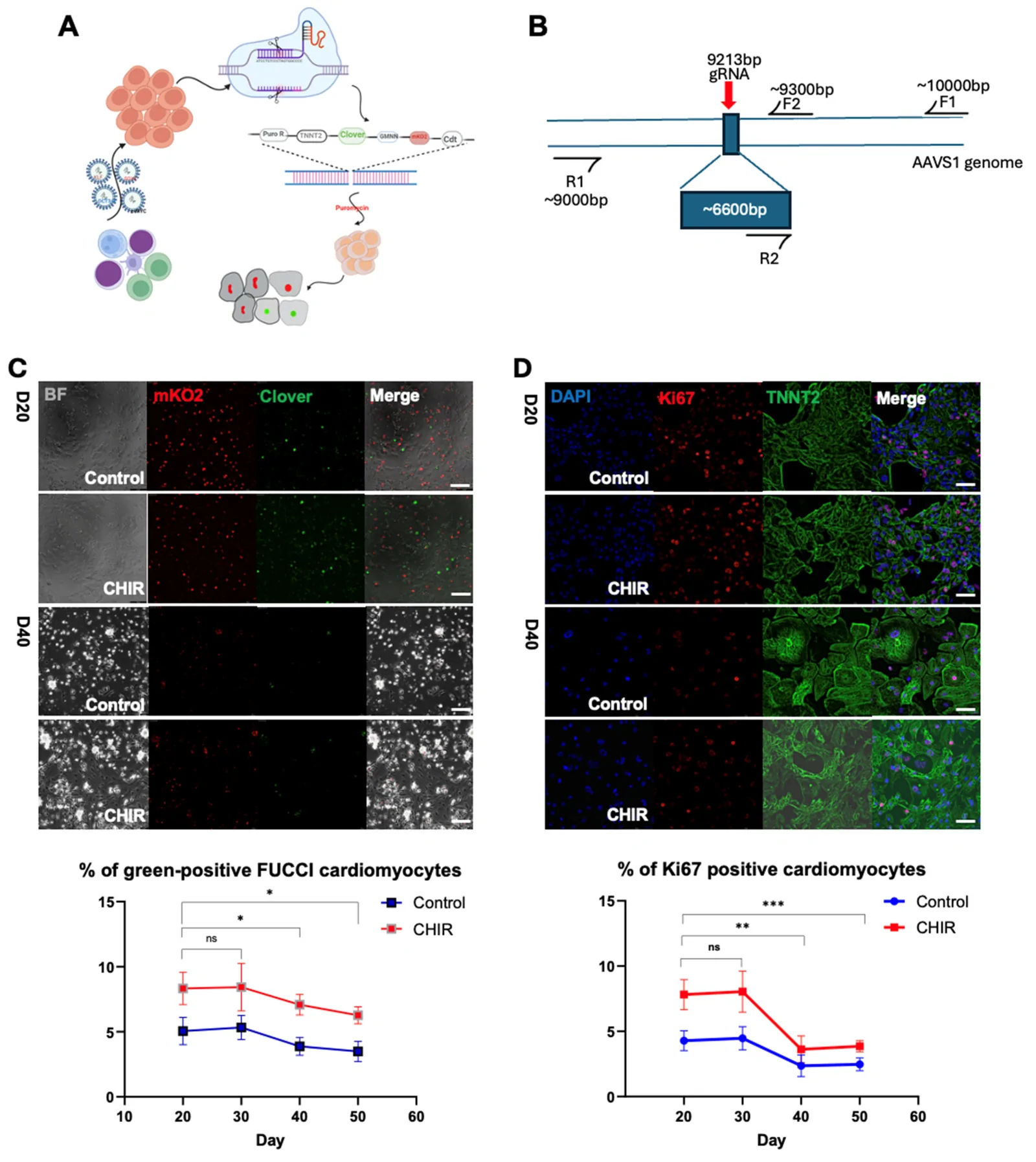
FUCCI and Ki67 analyses track cardiomyocyte cell-cycle activity during prolonged culture. (**A**) Schematic overview of the experimental workflow. Human iPSCs were engineered with a TNNT2-FUCCI reporter, differentiated into cardiomyocytes, and analyzed longitudinally to assess cell-cycle activity and maturation. (**B**) CRISPR/Cas9-mediated targeting strategy for insertion of the TNNT2-FUCCI reporter cassette into the AAVS1 safe-harbor locus. Primer locations and expected amplicon sizes used for genotyping are indicated. (**C**) Representative live-cell images of TNNT2-FUCCI cardiomyocytes cultured under control and CHIR-treated conditions at day 20 and day 40. Brightfield (BF), mKO2 (red), Clover (green), and merged channels are shown. Quantification of green-positive FUCCI cardiomyocytes across the maturation time course demonstrates maintenance of cell-cycle activity between days 20 and 30 followed by a decline at later stages. (**D**) Representative immunofluorescence images of day-20 and day-40 cardiomyocytes stained for Ki67 (red), TNNT2 (green), and DAPI (blue) under control and CHIR-treated conditions. Quantification of Ki67-positive TNNT2-positive cardiomyocytes across the maturation time course reveals a similar temporal pattern, with relatively stable cell-cycle activity between days 20 and 30 followed by reduced proliferation at later stages. Data are presented as mean ± SEM, with n = 3 independent biological replicates per time point. Statistical significance was assessed using two-way ANOVA followed by Šídák’s multiple-comparisons test for pairwise comparisons between time points within each treatment condition. Comparisons between control and CHIR-treated cells at individual time points were not included in the indicated statistical comparisons. A *p*-value < 0.05 was considered statistically significant. * *p* < 0.05, ** *p* < 0.01, *** *p* < 0.001; ns, not significant. Scale bar, 100 µm.

To determine when cell-cycle activity declines during prolonged culture of human iPSC-cardiomyocyte, we examined FUCCI reporter activity together with Ki67 immunostaining across sequential differentiation time points. A schematic of the experimental workflow and reporter targeting strategy is shown in Figure 1A,B. Correct reporter integration and clonal validation were confirmed by PCR genotyping and Sanger sequencing, and representative fluorescence images from the validated clone demonstrated detectable FUCCI reporter expression in differentiated cardiomyocytes (Figure S1A–C). Representative live-cell FUCCI images and Ki67/TNNT2 immunostaining images from days 20 and 40 are shown in Figure 1C,D, with quantification performed across days 20, 30, 40, and 50. In both control and CHIR-treated cultures, the percentage of FUCCI green-positive cardiomyocytes was similar at days 20 and 30 and decreased at days 40 and 50 (Figure 1C). CHIR-treated cultures generally showed a higher proportion of FUCCI-positive cells than controls, although the overall temporal pattern was similar. A comparable trend was observed by Ki67 staining, where the proportion of Ki67-positive TNNT2-positive cardiomyocytes remained relatively stable between days 20 and 30 but declined after 30 days (Figure 1D). Together, these data showed comparable cell-cycle activity at the day-20 and day-30 time points, with lower levels at days 40 and 50. To further assess whether early-stage cardiomyocytes progressed beyond cell-cycle entry into mitosis, we examined AURKB localization in day-19 TNNT2-positive cardiomyocytes. AURKB-positive mitotic structures were observed within a subset of TNNT2-positive cardiomyocytes (Figure S2), providing additional evidence that early-stage cardiomyocytes can progress through mitosis.

### 3.2. Transcriptional Analysis Identifies Early Suppression of Cell-Cycle Programs by Day 30 of Cardiomyocyte Differentiation

**Figure 2:**
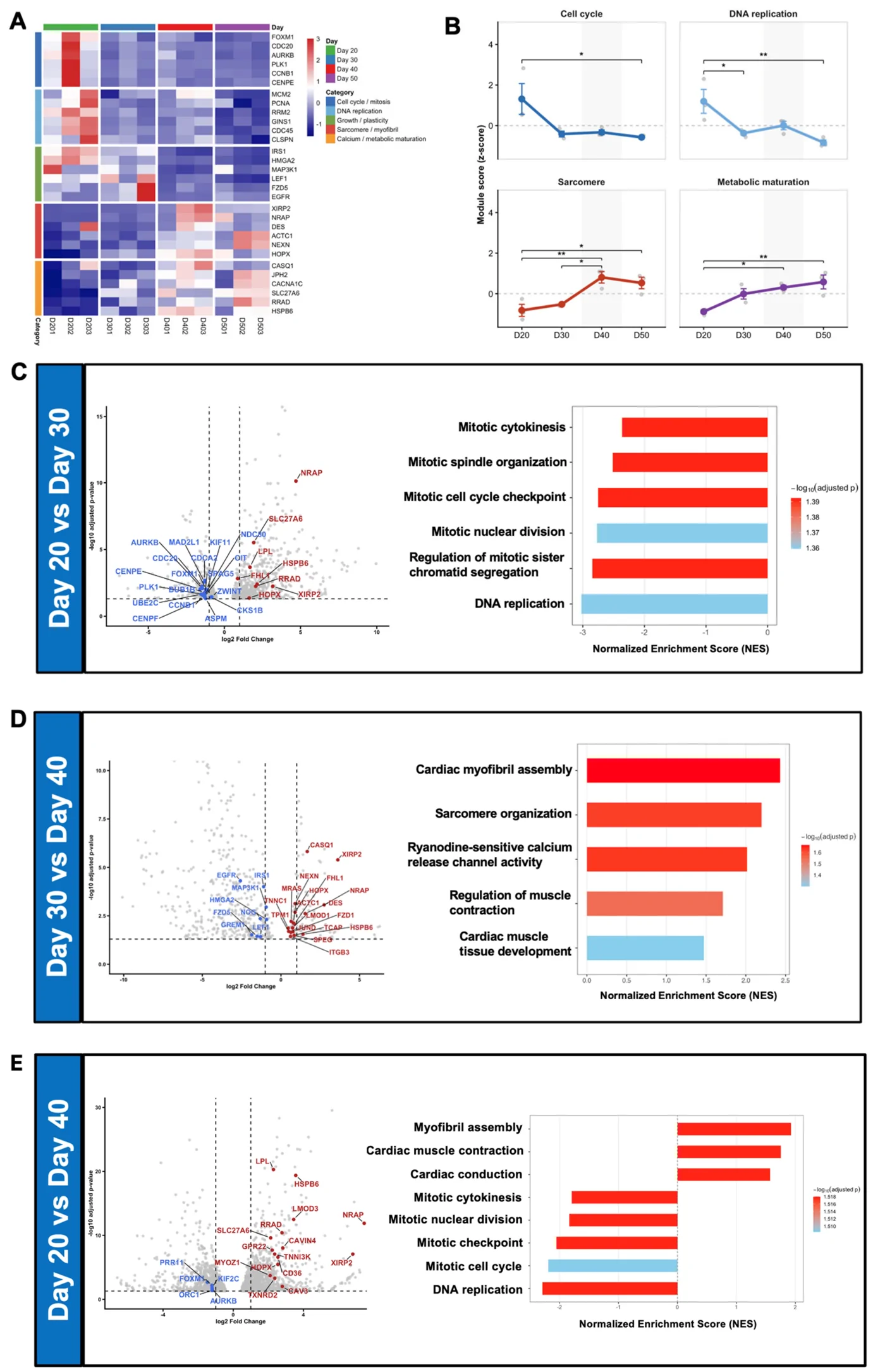
Temporal changes in cell-cycle and maturation-associated transcriptional programs during cardiomyocyte differentiation. (**A**) Heatmap of selected genes associated with cell-cycle progression, DNA replication, sarcomere organization, calcium handling, and metabolic maturation across days 20, 30, 40, and 50 of iPSC-cardiomyocyte differentiation. Heatmap values represent gene-wise row Z-scores calculated from normalized expression values, with the color scale indicating relative expression for each gene. Columns represent three independent cardiomyocyte differentiation replicates per time point (n = 3 biological replicates). (**B**) Module score analysis showing progressive decreases in cell-cycle and DNA-replication programs and increases in sarcomeric and metabolic maturation programs during prolonged cardiomyocyte culture. Statistical significance was assessed separately for each module using one-way ANOVA followed by Tukey’s multiple-comparisons test. Only significant pairwise comparisons are indicated. * *p* < 0.05, ** *p* < 0.01. (**C**) Differential expression and GOBP enrichment analyses comparing day-30 relative to day-20 cardiomyocytes. Mitotic and DNA-replication pathways were significantly reduced by day 30. (**D**) Differential expression and GOBP enrichment analyses comparing day-40 relative to day-30 cardiomyocytes. Maturation-associated pathways, including myofibril assembly, sarcomere organization, and muscle contraction, were enriched during this interval. (**E**) Differential expression and GOBP enrichment analyses comparing day-40 relative to day-20 cardiomyocytes. Combined suppression of proliferation-associated pathways and activation of maturation-associated pathways reflect the overall maturation trajectory. Differentially expressed genes in (**C**–**E**) were defined by |log2FC| > 0.5 and adjusted *p* < 0.05. Gene set enrichment analysis was performed using fgsea with Benjamini–Hochberg correction, with FDR < 0.05 considered significant. Positive and negative normalized enrichment scores (NES) indicate enrichment at the later and earlier time points, respectively. Representative significantly enriched pathways relevant to cell-cycle regulation, DNA replication, and cardiomyocyte maturation-associated transcriptional remodeling were selected for visualization.

To define the transcriptional changes that accompany loss of proliferative competence, we performed bulk RNA-seq on cardiomyocytes collected at days 20, 30, 40, and 50. A curated heatmap of genes associated with proliferation, growth-related signaling, and cardiomyocyte maturation revealed two major temporal patterns (Figure 2A). Genes involved in cell-cycle progression and DNA replication were highest at day 20 and were reduced by day 30, whereas genes linked to sarcomeric structure, calcium handling, and metabolic remodeling increased at days 40 and 50. These temporal patterns were supported by curated module scoring across the time course, which showed that cell-cycle and DNA-replication modules declined early, while sarcomere and metabolic remodeling-associated modules increased progressively as differentiation advanced (Figure 2B).

We next examined differential gene expression between days 20 and 30 to define the changes in cell-cycle transcription during this interval. Multiple canonical mitotic regulators, including FOXM1, AURKB, CENPE, CENPF, PLK1, UBE2C, CCNB1, NDC80, and MAD2L1, were decreased at day 30 (Figure 2C). These genes regulate interconnected components of the mitotic program, including mitotic entry and progression, spindle and kinetochore function, chromosome segregation, and cytokinesis [12,13,14]. FOXM1, in particular, coordinates a transcriptional program required for mitotic progression, including regulation of CCNB1, PLK1, AURKB, and CENPF. In contrast, several genes associated with structural and metabolic aspects of cardiomyocyte maturation, including NRAP, XIRP2, HSPB6, RRAD, and SLC27A6, increased at day 30 (Figure 2C). NRAP functions as a scaffold during cardiomyocyte myofibril assembly [15], while SLC27A6, a cardiac fatty-acid transporter, has been linked to metabolic maturation of human iPSC-derived cardiomyocytes [16]. To determine whether these gene-level changes reflected coordinated biological programs, we performed GO biological process enrichment analysis. Pathways reduced by day 30 relative to day 20 included DNA replication, mitotic cell-cycle checkpoint, mitotic spindle organization, mitotic nuclear division, cytokinesis, and regulation of sister chromatid segregation (Figure 2C). Together, these results suggest that proliferation-associated transcriptional programs were significantly reduced by day 30, preceding the significant decrease in the proportion of cell-cycle active cardiomyocytes detected at day 40 (Figure 1C,D).

### 3.3. The Day-30-to-Day-40 Transition in Cardiomyocyte Differentiation Is Characterized by Increased Maturation-Associated Transcriptional Programs

Since the measurable decline in cell-cycle activity occurred after day 30 (Figure 1C,D), we next examined the transcriptional changes between days 30 and 40. Unlike the transition between day-20 and day-30 cardiomyocytes, the day-30-to-day-40 interval was not dominated by another broad decrease in canonical mitotic genes. Instead, several genes associated with cardiomyocyte structural and functional maturation, including XIRP2, NRAP, NEXN, HOPX, CASQ1, FHL1, TCAP, and HSPB6, were increased at day 40 (Figure 2D). Among these, HOPX has been identified as a regulator of late-stage maturation programs in human pluripotent stem cell-derived cardiomyocytes, supporting the interpretation that this interval reflects increasing activation of maturation-associated transcriptional programs. Consistent with this shift, GO biological process analysis identified enrichment of cardiac myofibril assembly, sarcomere organization, ryanodine-sensitive calcium-release channel activity, regulation of muscle contraction, and cardiac muscle tissue development during the transition from day 30 to day 40 (Figure 2D). Myofibrillar and sarcomeric organization, together with development of specialized calcium-handling and contractile properties, are characteristic features of cardiomyocyte maturation [17,18]. Thus, whereas the day-20-to-day-30 interval was characterized by significant reductions in DNA-replication and mitotic programs, the day-30-to-day-40 interval was characterized by increased expression of structural, contractile, and calcium-handling programs associated with cardiomyocyte maturation.

To examine the overall transcriptional differences between days 20 and 40, we directly compared the transcriptomic profiles at these two time points. This analysis showed the combined signature of reduced proliferation-associated pathways together with increased cardiac structural and contractile programs (Figure 2E), consistent with the overall maturation-associated transcriptional trajectory observed across the time course. As an additional endpoint comparison, day-20 versus day-50 analysis showed a similar overall pattern, with reduced proliferation-associated programs together with enrichment of maturation-related structural and contractile pathways, supporting continued progression of the maturation-associated transcriptional remodeling at later stages (Figure S1D).

Taken together, these data support a staged pattern of transcriptional remodeling during prolonged iPSC-derived cardiomyocyte culture. Cell-cycle and DNA-replication programs were significantly reduced by day 30, preceding the significant decrease in FUCCI and Ki67-based measures of cell-cycle activity at day 40. The subsequent day-30-to-day-40 interval was characterized by increased expression of structural, contractile, and calcium-handling programs associated with cardiomyocyte maturation. Thus, reduced cell-cycle transcription and increased maturation-associated transcription were observed during different intervals of the time course, with cell-cycle programs decreasing between days 20 and 30 interval and maturation-associated programs increasing predominantly between days 30 and 40.

## 4. Discussion

Our findings suggest that cardiomyocyte cell-cycle withdrawal occurs progressively during prolonged culture rather than as a single event. The early reduction in cell-cycle and DNA-replication programs despite continued cell-cycle activity at day 30 may indicate an intermediate state during the transition toward cell-cycle withdrawal. Previous studies have shown that hiPSC-derived cardiomyocytes can remain capable of cell-cycle activity while also undergoing changes associated with maturation, and that cell-cycle activity does not always result in productive cell division [8]. Therefore, the reduction in cell-cycle-associated transcription observed at day 30 may represent an early transition toward cell-cycle withdrawal, while some cardiomyocytes remain cell-cycle-active. Importantly, FUCCI and Ki67 identify cells with cell-cycle activity but do not distinguish completed cytokinesis from other outcomes such as multinucleation or polyploidization, which should be considered when interpreting the later decrease in these markers [8]. The transcriptional changes observed during this transition are consistent with progressive activation of maturation-associated gene programs. Cardiomyocyte maturation involves progressive changes in sarcomere organization, contraction, calcium handling, metabolism, and gene expression [18]. Previous studies have shown that maturation-associated transcriptional programs develop progressively during differentiation of human PSC-derived cardiomyocytes. In particular, HOPX has been identified as an important regulator of later cardiomyocyte maturation, with HOPX-associated gene programs linked to cardiac structure and function [19,20]. More recent work has also shown that PRDM16 can influence the balance between proliferation and maturation in hiPSC-derived cardiomyocytes, further supporting the close relationship between these processes [21]. Together, these studies support a close association between declining cardiomyocyte cell-cycle activity and the acquisition of maturation-associated characteristics during differentiation, although the present study does not establish a direct causal relationship between the two processes.

Several limitations should be considered when interpreting these findings. The RNA-seq analysis was performed using bulk populations and therefore cannot determine whether the transcriptional changes occur within the same cells that subsequently lose cell-cycle activity. In addition, RNA-seq measures transcript abundance and does not directly establish corresponding changes in protein abundance. Differences in protein translation, stability, and turnover may result in temporal differences between transcriptional changes and their functional consequences. Although the decline in cell-cycle activity was independently supported by FUCCI and Ki67 measurements, protein-level validation of specific cell-cycle regulators identified by RNA-seq was not performed. Therefore, the relationship between the observed transcriptional changes and subsequent changes in cell-cycle activity should be interpreted cautiously. Furthermore, the FUCCI experiments were performed using a single TNNT2-FUCCI iPSC clone derived from one donor, while the bulk RNA-seq experiments were performed using control iPSC-derived cardiomyocytes without the FUCCI reporter; therefore, potential donor- and clone-specific effects cannot be excluded. Validation in additional independent iPSC lines and reporter clones will be important to establish the generalizability of these findings. The four time points used in this study also do not define the exact timing of the transition between these states. Finally, FUCCI and Ki67 measurements indicate cell-cycle activity but do not directly demonstrate completed cell division. The transcriptional changes observed in this study were not accompanied by direct measurements of functional maturation, such as beating kinetics, calcium transients, mitochondrial function, or metabolic substrate utilization. Therefore, the observed changes should be interpreted as maturation-associated transcriptional remodeling rather than evidence of complete functional maturation. Future studies using additional time points, single-cell analysis, and direct measurements of cytokinesis and cardiomyocyte function will help further define the relationship between cell-cycle withdrawal and maturation.

## 5. Conclusions

By combining live-cell FUCCI imaging with Ki67 analysis and transcriptomic profiling, we show that changes in cell-cycle-associated transcription, cell-cycle activity, and maturation-associated programs occur at distinct stages during prolonged cardiomyocyte culture. These findings provide a temporal framework for distinguishing changes in cell-cycle regulation from the acquisition of maturation-associated transcriptional programs during prolonged hiPSC-CM culture.

## Data Availability

The data supporting the findings of this study are available from the corresponding author upon reasonable request. Bulk RNA-sequencing data have been deposited in the NCBI Gene Expression Omnibus (GEO) under accession number GSE349281.

## Supplementary Materials

The following supporting information can be downloaded at: https://www.mdpi.com/article/10.3390/cells15211936/s1.
