# Supplementary material for: Live-Cell Monitoring and Transcriptomic Profiling Identify Distinct Temporal Patterns of Cell-Cycle Activity and Maturation in Human iPSC-Derived Cardiomyocytes
Source: Cells. 2026 Oct 24;15(21):1936. doi: 10.3390/cells15211936 (PMC13761616; doi:10.3390/cells15211936)
Supplement: Supplementary file 1 [file cells-15-01936-s001.zip › cells-4547297-supplementary.pdf]

Supplementary Figure S1

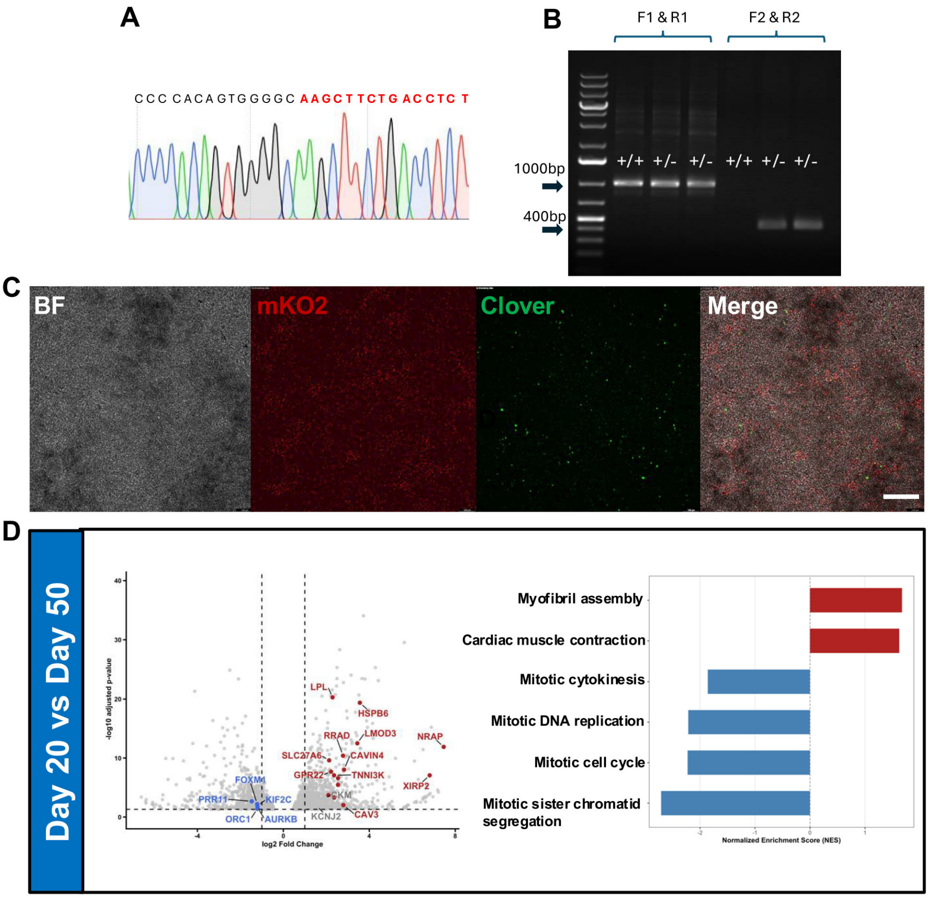

Figure S1. **Generation and validation of the TNNT2-FUCCI reporter line and extended transcriptomic comparison.** (A) Sanger sequencing chromatogram confirming correct integration of the TNNT2-FUCCI reporter construct at the targeted genomic locus.(B) PCR-based genotyping of monoclonal TNNT2-FUCCI iPSC lines using primer pairs F1/R1 and F2/R2 to identify correctly targeted clones.(C) Representative live-cell fluorescence images of TNNT2-FUCCI cardiomyocytes showing brightfield (BF), mKO2 (red), Clover (green), and merged channels.(D) Differential expression and GOBP enrichment analyses comparing Day 20 and Day 50 cardiomyocytes. Maturation-associated pathways, including myofibril assembly and cardiac muscle contraction, were enriched, whereas DNA replication, mitotic cell-cycle progression, cytokinesis, and sister chromatid segregation pathways were reduced.

Supplementary Figure S2

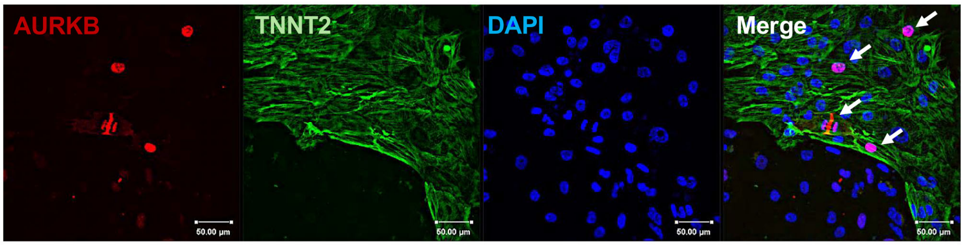

Figure S2. **AURKB staining in early-stage hiPSC-derived cardiomyocytes.** Representative immunofluorescence images of Day 19 hiPSC-derived cardiomyocytes stained for AURKB (red), cardiac troponin T (TNNT2; green), and DAPI (blue), with the merged image shown on the right. White arrowheads indicate AURKB-positive cells within the TNNT2-positive cardiomyocyte population, supporting progression of a subset of cardiomyocytes through mitosis. Scale bar, 50 μm.
